# Newcastle Disease Virus Induces Profound Lymphoid Depletion with Different Patterns of Necroptosis, Necrosis, and Oxidative DNA Damage in Bursa, Spleen, and Other Lymphoid Tissues

**DOI:** 10.3390/pathogens13080619

**Published:** 2024-07-26

**Authors:** Mohammad Rabiei, Milton M. McAllister, Natalie R. Gassman, Kevin J. Lee, Sydney Acton, Dieter Liebhart, Wai Yee Low, Farhid Hemmatzadeh

**Affiliations:** 1 School of Animal and Veterinary Sciences, The University of Adelaide, Adelaide, SA 5371, Australia; 2 Gastro-Intestinal Viral Oncology Group, Ingham Institute for Applied Medical Research, Liverpool, Sydney, NSW 2170, Australia; 3 South Western Sydney Clinical School, University of New South Wales, Liverpool, Sydney, NSW 2170, Australia; 4 Mitchell Cancer Institute, University of South Alabama, Mobile, AL 36604, USA; 5 Clinical Department for Farm Animals and Food System Science, Clinical Centre for Population Medicine in Fish, Pig and Poultry, University of Veterinary Medicine, 1210 Vienna, Austria; 6 The Davies Research Centre, School of Animal and Veterinary Sciences, The University of Adelaide, Adelaide, SA 5371, Australia

**Keywords:** Newcastle disease virus (NDV), lymphoid depletion, necroptosis, apoptosis, repair assisted damage detection (RADD), oxidative damage, immunosuppression

## Abstract

This study delves into the pathogenesis of virulent genotype VII strains of the Newcastle disease virus (NDV), focusing on experimentally infected birds. Predominant and consistent lesions observed include bursal atrophy and extensive depletion of all lymphoid tissues. Immunohistochemistry (IHC) analysis, targeting apoptosis (Caspase-3), necroptosis (MLKL), and NDV markers, indicates that bursal atrophy is linked to a non-apoptotic programmed cell death pathway known as “necroptosis”. Repair assisted damage detection (RADD) of the bursa reveal oxidative DNA damage patterns consistent with programmed cell death, aligning with MLKL expression. Contrastingly, in the spleen, our findings suggest that necrosis (non-programmed cell death) predominantly contributes to lymphoid depletion. This conclusion is supported by evidence of karyorrhexis, fibrinous inflammation, RADD analyses, and IHC. Moreover, in addition to being pathogenic in its own right, NDV caused extensive and rapid lymphoid depletion that should be expected to contribute to profound immunosuppression. The elucidation of necroptosis in NDV-infected chickens provides a good rationale to investigate this mechanism in other paramyxoviral diseases such as human measles.

## 1. Introduction

The Newcastle disease virus (NDV), a member of the Paramyxoviridae family, poses a global threat to poultry and wild birds due to its worldwide distribution and ability to infect various avian species [1]. Infection of birds with highly virulent strains of NDV results in immunosuppression, which at least in part results from profound depletion of lymphoid tissues [2,3].

NDV has also been extensively studied in human tumour cells for its potential role as an oncolytic therapeutic agent [4]. Existing studies have explored the relationship between programmed or non-programmed cell death and NDV or other paramyxoviruses [4,5,6].

Viruses are obligate intracellular infectious agents that use the host cell transcriptome machinery to replicate the viral genome and produce viral protein [7]. Virus–host–cell interactions involve DNA transactions, including the induction of DNA damage. Viruses, including NDV, interact with the host’s DNA damage response (DDR) machinery, inducing DNA damage and potentially leading to programmed cell death. The host cell uses DDR signalling to induce cell cycle arrest to mitigate damage, promote repair, or induce cell death. Significant DNA damage levels can lead DDR proteins to start apoptotic programmed cell death to preserve host genomic integrity [8]. Programmed cell death, including apoptosis and necroptosis, are critical biological processes that regulate cellular homeostasis and response to stress [9]. Necrosis is unprogrammed, unregulated cell death that is caused by injuries leading to failure of essential cellular processes such as energy production, protein synthesis, or lysis of cell membranes [10].

Apoptosis, a caspase-mediated programmed cell death pathway, is a hallmark of cytotoxicity in human tumour cell lines infected in vitro with Newcastle disease virus (NDV), triggering extrinsic and intrinsic apoptotic pathways [4]. Necroptosis, a noncaspase-dependent and precisely regulated mechanism of cell death, has been observed in oncolytic experiments of NDV using human tumour cells [5,6]. However, the relationship between necroptosis and Newcastle disease in birds, or with other naturally occurring paramyxoviral diseases such as measles, remains unexplored.

Repair assisted damage detection (RADD) is a methodology for detecting a broad spectrum of DNA lesions, including oxidative, crosslinks, uracils, abasic sites, and strand breaks [11]. Oxidative RADD (oxRADD) detects the subset of RADD lesions specifically caused by oxidation. Detection and measurement of DNA lesions using both the full broad-spectrum RADD and oxRADD allows the specific quantification of oxidative lesions from the total DNA lesion load with the tissue. These powerful techniques are capable of revealing new information about the type and extent of DNA damage and DNA repair occurring after various cellular insults, including infection with DNA and RNA viruses [8,12].

The purpose of this study is to investigate highly virulent NDV in experimentally infected chickens in order to evaluate cell death patterns and to explore the relationship of virus and cell death types with DNA damage patterns. This study includes exploration of necroptosis, a programmed cell death pathway not previously investigated in NDV-infected chickens or in other paramyxoviruses such as measles. We use immunohistochemistry (IHC) to detect the cell death markers caspase-3 and mixed lineage kinase domain-like protein (MLKL) to help differentiate between the apoptosis, necroptosis, and necrosis of lymphoid tissues. To our knowledge, this study is the first to investigate MLKL expression and to perform repair assisted damage detection (RADD) analyses in NDV-infected birds.

## 2. Material and Methods

### 2.1. Animal Experiments

The animal experiments were conducted at the Indonesian Research Centre for Veterinary Science (Bbalitvet) in Bogor, Indonesia, following ethical approval from the research and animal ethics committee of Bbalitvet Institute and the University of Adelaide (reference number AH/2015/003). Adhering to the National Health and Medical Research Council (NHMRC) of Australia guidelines and Animal Research Reporting of In Vivo Experiments (ARRIVE) guidelines 2.0, an experienced veterinarian managed the challenge experiment. Nineteen specific-pathogen-free (SPF) broiler Ross chickens, sourced from Caprifarmindo Laboratories (Bandung, Indonesia), were randomly divided into two groups and housed in negative-pressure isolators at biosafety level 3 (BLS3).

### 2.2. Virus and Challenge

Our study employed the genotype VII Mega strain of NDV, isolated in 2015 from chicken brains in West Java, Indonesia. Characterised as high virulence viruses by OIE criteria [13], in prior analyses the Mega strain virus had a mean death time (MDT) between 67 h and 60 h (accession number MN688613). For highly pathogenic NDVs, researchers typically employ infectious challenges within a range of 10^1.5^ to 10^4.2^ embryo infectious doses (EIDs_50_) [14,15]. The present infectious challenge involved inoculating nine birds with 10^2^ EIDs_50_ of NDV at 35 days of age, administered intraocularly and intratracheally. Due to the unexpectedly rapid onset of severe clinical signs and mortality, all remaining birds in the infected group were euthanized on the second day after challenge (when 37 days old), while negative controls were euthanized as originally scheduled (when 38 days old). Necropsy examinations were performed on the day of death, and tissue specimens for histopathology, immunohistology, and repair assisted damage detection (RADD) analyses were fixed in 10% neutral buffered formalin.

### 2.3. Site Detection of NDV in Chicken Tissues

From each chicken, tissue samples were taken from the spleen, bursa of Fabricius, brain, liver, and lung. Viral RNA was extracted from tissue samples using a QIAamp Viral RNA Mini kit (Qiagen, Louisville, KY, USA) and quantified using a NanoDrop 1000 Spectrophotometer (Thermo Fisher Scientific, Carlsbad, CA, USA). An amount of 5 μL of extracted RNA was converted to cDNA using a QuantiTect Reverse Transcription Kit (Qiagen, Louisville, KY, USA) as per the manufacturer’s instructions. A conventional PCR method was performed for detection of the NDV-fusion protein using forward: 5′-ATGGGCYCCAGACYCTTCTAC-3′, and reverse: 5′-CTGCCACTGCTAGTTGTGATAATCC-3′ primers, generating a 535 bp amplicon [16].

### 2.4. Haematoxylin and Eosin Staining and Immunohistochemistry

Tissue samples of the spleen, bursa of Fabricius, brain, kidney, lung, caecum, small intestine, pancreas, and proventriculus from the negative control and NDV-challenged birds were collected in 10% neutral buffered formalin (Sigma-Aldrich, Sydney, NSW, Australia). Thymus was collected when visualised but was not always apparent. The tissues were processed routinely within a week of fixation and embedded in paraffin wax. Sections (5 μm) were stained by Hematoxylin and Eosin (HE) or were subjected to immunohistochemical (IHC) staining for caspase-3 and MLKL antigens and for the NDV HN antigen using a Dako Omnis Autostainer system. The Dako machine’s heat-induced method was applied for antigen retrieval using the EnVision FLEX Target Retrieval Solution at pH 6.0 for 30 min. Antigens were labelled with primary anti-NDV HN monoclonal antibody (Table 1) and visualised using DAKO EnVision FLEX/HRP (DAB). Double staining was performed using anti-cleaved caspase-3 antibody ab4051 or anti-MLKL (non-phosphorylated) antibody MABC604 of the tissue samples and visualised by EnVision FLEX Magenta Red (Agilent, Santa Clara, CA, USA). Sections were counterstained with Mayer haematoxylin for 30 s before applying coverslips with DPX mounting media.

### 2.5. Lesion Scoring of Lymphoid Tissues

H&E slides of the spleen, bursa of Fabricius, caecal tonsils, or other areas of gut-associated lymphoid tissue (GALT) and thymus were examined and scored according to the following scale: 1 = normal dense lymphoid tissue, 2 = less dense lymphoid tissue, 3 = equivocal lymphoid lesion suggestive of lymphoid depletion or increased apoptotic bodies, 4 = obvious lymphoid depletion with numerous apoptotic or karyolytic cells, and 5 = massive lymphoid depletion with apoptotic appearance and/or necrotic appearance with karyolysis, often with fibrinous exudate. For each bird, a single average or composite lesion score was calculated for all lymphoid tissues.

### 2.6. DNA Assessment Using Repair Assisted Damage Detection (RADD)

DNA damage was detected within the virally infected tissues using the RADD assay. Formalin-fixed paraffin-embedded (FFPE) tissues were sectioned and assessed for a broad spectrum of DNA lesions, i.e., abasic sites, oxidative lesions, pyrimidine dimers, uracils, deamination events, and strand breaks, which were labelled using the RADD cocktail (full RADD, Table 2). Additionally, oxidative lesions were specifically examined using a cocktail of FPG, EndoIV, and EndoVIII (oxRADD). Full RADD and oxRADD signals occur predominantly within the nuclei of cells, indicating genomic DNA damage [11].

The bursa of Fabricius was chosen to investigate RADD for its suitability in analysing different types of DNA damage associated with NDV infection. Because the histopathological and IHC procedures were performed before the RADD procedures, some of the tissues had been consumed, but enough bursal specimens remained to compare 8 infected with 7 uninfected birds by RADD and then 7 infected with 7 uninfected birds by oxRADD. Tissues were sectioned in 5 μm thick slices and mounted on Superfrost glass slides (Fisher Scientific, Hampton, NH, USA). The slides were placed on a heat block set at 65 °C and incubated for 8 min to melt the paraffin. The slides were then placed directly in 100% xylene and incubated twice for 10 min each. The slides were rehydrated in water through sequential incubations in ethanol and water mixtures. Specifically, the slides were incubated for 5 min each in sequential order of 100% ethanol–0% water; 70% ethanol–30% water; 50% ethanol–50% water; 30% ethanol–70% water; 0% ethanol–100% water. The rehydrated slides were then placed in glass Coplin jars with 200 mL of 10 mM sodium citrate in water and microwaved twice for 2.5 min at 1200 watts until the solution reached 47 °C for antigen retrieval. The slides were allowed to cool in water for 2 min. The slides were briefly dried, and tissue samples were outlined with a hydrophobic barrier using a PAP pen.

The lesion removal cocktail (Table 2) was added to each tissue sample and incubated for 1 h at 37 °C. For the full RADD broad-spectrum lesion removal cocktail, all enzymes listed in Table 2 were included. For oxidative lesions only (oxRADD), T4 PDG and UDG were omitted from the lesion removal cocktail and replaced with water. A gap-filling solution (Table 2) was then added directly to the lesion removal solution and incubated for another hour at 37 °C. The slides were washed three times in phosphate-buffered saline (PBS) for 5 min each and blocked in 2% BSA in PBS for 30 min at room temperature (RT). Anti-digoxigenin (Dig) antibody (Abcam; #ab420 clone 21H8, Abcam, Cambridge, MA, USA) was incubated at 1:250 dilution in 2% BSA in PBS at 4 °C overnight. As a negative control for the Dig antibody, an extra slide processed with the full RADD enzyme cocktail was incubated with mouse IgG isotype control antibody (Cell Signalling 5415, clone G3A1, Cell Signaling Technology, Danvers, MA, USA) at a dilution of 1:625 at 4 °C overnight. This dilution factor matched the µg of anti-Dig antibody used per 100 µL. The next day, the slides were washed three times in PBS for 5 min each, and Alexa Fluor 546 goat anti-mouse secondary antiserum (Invitrogen, Carlsbad, CA, USA) was incubated at 1:400 dilution in 2% BSA in PBS for 1 h at R.T. Hoescht 33342 was added at a final dilution of 1:1000 for 15 min at RT to stain the nuclei. The sliddried andshed three times in PBS for 5 min each, briefly dried, and mounted with coverslips using ProLong Gold Antifade reagent (Invitrogen). The slides were allowed to dry overnight in the dark at RT and visualised using a Nikon A1R confocal microscope or stored at 4 °C until analysis.

### 2.7. RADD Image Acquisition

Following protocols established by Lee et al. (2019), all images for RADD were acquired using a Nikon A1r scanning confocal microscope with a Plan-Apochromat 10×/0.5 objective [11]. Image acquisition settings were obtained by imaging the full RADD samples for tissues and identifying gain settings that limited the number of saturated pixels. These imaging conditions were then used for all tissue imaging, allowing for direct comparisons and analysis between tissues. For large tissue sections, images were first mapped using the Acquire Large Image acquisition tool in the Nikon Elements software (NIS-Elements AR 4.51.00), acquired using the 10× objective, and stitched post-acquisition. The software maps the X-Y positions of individual images within the tissue slice, which are then acquired individually at 10×, 1024 × 1024 resolution for further analysis. The fluorescent intensity for each 1024 × 1024 segment is recorded after a binary threshold is applied. Each tissue section required between 4 and 10 1024 × 1024 images to cover the tissue section completely, depending on the area of the sectioned tissue.

### 2.8. Statistical Analyses

Composite lymphoid lesion scores were compared between infected and negative control birds using Student’s *t*-test. For RADD and oxRADD the fluorescence intensity was captured for each 1024 × 1024-pixel segment in arbitrary units and the mean fluorescence intensity for each tissue section was calculated from the combined segments. Mean fluorescent values were compared between infected and uninfected groups using Student’s *t*-test.

## 3. Results

### 3.1. Clinical Signs and Gross Lesions

All birds in the challenged group showed severe clinical signs of ND and were severely depressed. Clinical signs included reddened and swollen conjunctiva, dehydration, ruffled feathers, pale comb, weight loss, anorexia, hunched posture, reduced activity, squinting or closed eyelids, recumbency, and death. Three of the infected birds were found dead one day after inoculation with the virus, and two others were found dead on the second day (when the birds were 37 days old), when it was decided to euthanize the remaining infected cohort ahead of schedule for humane reasons. All birds in the uninfected control group were euthanized on day 3 as originally planned (when the birds were 38 days old). All carcasses were necropsied on the day of death in a glovebox using BCL3 procedures; this limited our ability to take quality photographs of gross lesions. Gross lesions were observed only in infected birds. The most pronounced and consistent gross lesions were bursal atrophy and indistinct mottling of spleen. Other gross lesions included thymic atrophy and small numbers of petechial haemorrhages in the mucosa of the proventriculus. These findings demonstrate that severe ND was induced, while the uninfected birds remained healthy.

### 3.2. Detection of Virus in Tissues by PCR

PCR detection of NDV DNA was positive in nine of nine infected birds from the spleen, bursa, liver and lung, and in one of nine brains, while PCR results were uniformly negative in all 10 uninfected control birds. The results of IHC for viral antigen were concordant, as reported below.

### 3.3. Histopathology

**Figure 1:**
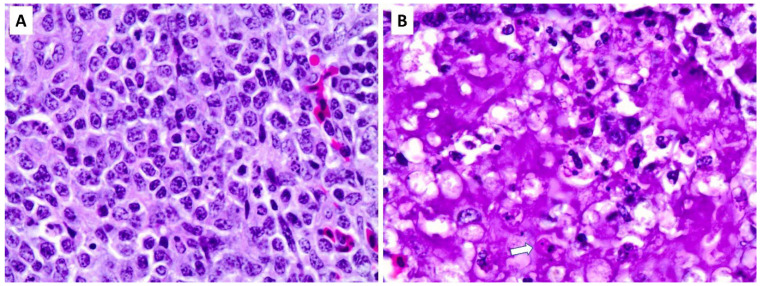
Histopathological lesions in the spleen. The spleen of an uninfected control bird (**A**) has normal histological and cellular morphology. An NDV-infected bird (**B**) has profound lymphoid depletion with remnant hypereosinophilic cell bodies (white arrow), condensed nuclear debris (karyorrhexis), and an abundant exudate of pink (eosinophilic) fibrin. This appearance is typical for uncontrolled cell death (necrosis) and secondary inflammation.

The 10 birds in the uninfected control group had no significant histopathological lesions. Composite lesion scores for lymphoid tissues of each bird in this group ranged between 1.16 and 1.5 on a five-point scale (i.e., densely cellular healthy lymphoid tissues). All nine infected birds had severe lesions in lymphoid tissues in the bursa, spleen, thymus, caecal tonsil, and other GALT and bronchial-associated lymphoid tissues (BALTs), with composite lesion scores ranging between 4.17 and 5, respectively, on a five-point scale. The T-test revealed a highly significant difference (*p* < 0.001) in lesion scores between infected birds (mean score: 4.585) and the control group (mean score: 1.33). The spleens had profound lymphoid depletion with a combination of nuclear pyknosis and karyorrhexis. Most spleens also had moderate to abundant fibrinous exudate that prevented tissue collapse secondary to loss of white pulp (Figure 1).

**Figure 2:**
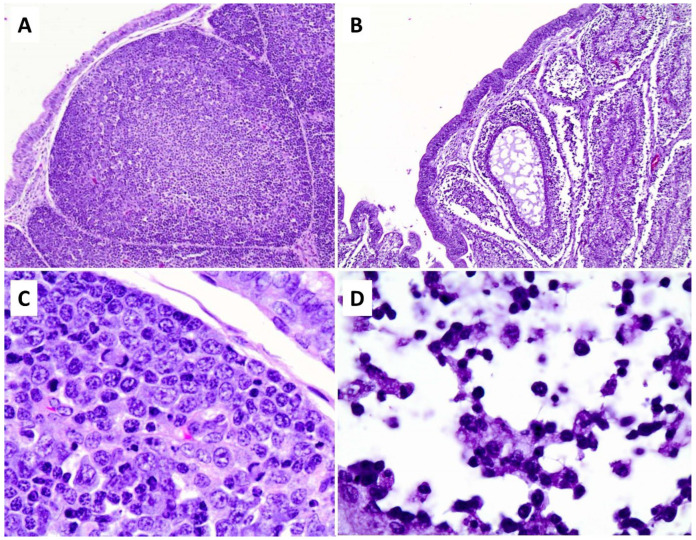
Histopathological lesions in bursa of Fabricius. Top row: Identically magnified views of bursa of Fabricius. In comparison with an uninfected control bird (**A**), bursal follicles of an NDV-infected bird (**B**) are small, collapsed, and hypocellular. Bottom row: Higher magnifications of follicular cortex. Lymphocytes in an uninfected bird (**C**) appear healthy. In an infected bird (**D**), lymphocytes are depleted and remnant nuclei have dark condensed nuclei (pyknosis), but there is no fibrinous exudate or other evidence of inflammation. This appearance is consistent with programmed cell death.

Each bursa of Fabricius of the infected birds was visibly atrophied and had severe lymphoid depletion with almost complete loss of medullary lymphocytes, moderate to severe loss of cortical lymphocytes, notable reduction in size of follicles and plicae, and relative visual enhancement of the unaffected internal epithelium. In contrast with the spleen, the lymphocytes in the bursa only showed pyknotic-like nuclear condensation without evidence of karyorrhexis, and there was no fibrinous exudate or other evidence of inflammation (Figure 2).

Thymus was often difficult to observe grossly to enable collection from infected birds, so only three infected thymus were examined. Lymphoid depletion with nuclear karyorrhexis and fibrinous exudate occurred in medullary centres and extended well into lobule cortices, with a sparing of about 20% of the lymphoid tissue located at the periphery of the cortical regions. Caecal tonsils and other GALT in the proventriculus, small intestine, and colon were also profoundly depleted in all nine of the NDV-infected birds, often associated with vascular congestion or mild mucosal or submucosal haemorrhage. BALT was depleted or necrotic in lungs. Apart from the depletion of GALT, gastrointestinal lesions were inconsistent and minor, consisting of congestion or haemorrhage associated with lymphoid depletion. Mild to moderate lesions were observed in the liver of all nine infected birds variably including scattered individual necrotic hepatocytes, small, scattered foci of necrosis, patchy vacuolar degeneration of hepatocytes, vascular thrombosis, focal fibrin exudate, and bile stasis. Two birds had small foci of necrosis in the exocrine pancreas.

Histopathological lesions were observed in two hearts of infected birds, including one with a cellular thrombus and one with diffuse myocardial oedema. In the kidneys, focal tubular epithelial necrosis or autolysis was observed in two birds that were found dead on the second day after infection, and one bird had ectasia of scattered renal tubules which contained droplets and cell debris in their lumens. Only one bird had brain lesions, consisting of focal venous thrombosis and mild scattered oedema. In short, histopathological lesions were consistent with induction of severe ND in the infected cohort.

### 3.4. Immunohistochemical Results

**Figure 3:**
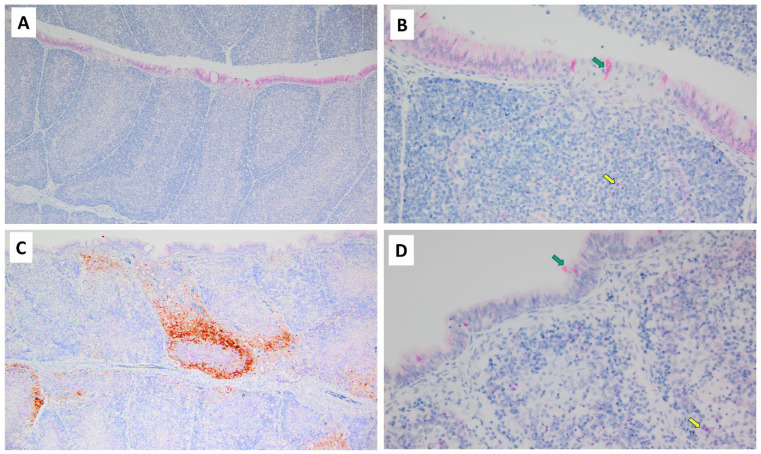
Immunohistochemistry of bursa of Fabricius for caspase-3 (red stain) and NDV HN (brown stain). (**A**,**B**) Two magnifications of a bursa from an uninfected chicken. There is no evidence of viral antigen, but panel B shows that sparsely scattered individual lymphocytes (yellow arrow) and mucosal epithelial cells (green arrow) exhibit caspase-3 antigen. (**C**,**D**) Two magnifications of a bursa from an infected chicken. Panel C shows unevenly distributed staining for viral antigen, with individual follicles either showing heavy staining or almost no staining. In (**D**), the bursal tissue has only modest background expression of caspase within sparsely dispersed individual lymphocytes (yellow arrow) and mucosal epithelial cells (green arrow), similar to caspase expression in uninfected chickens.

Immunohistological staining for the NDV HN antigen was negative in all tissues of all 10 uninfected control birds. All nine infected birds had some degree of NDV HN antigen staining in lymphoid and other tissues. The pattern observed in the bursa of Fabricius was heavy viral antigen staining of follicles that were unevenly distributed among negatively staining follicles (Figure 3), despite the fact that profound lymphoid depletion was diffusely distributed throughout each bursa (Figure 2B). The most diffuse and dark staining for viral antigen was observed in the three thymuses that were collected from infected birds. Spleens had less dense staining for viral antigen than did thymuses and bursae.

**Figure 4:**
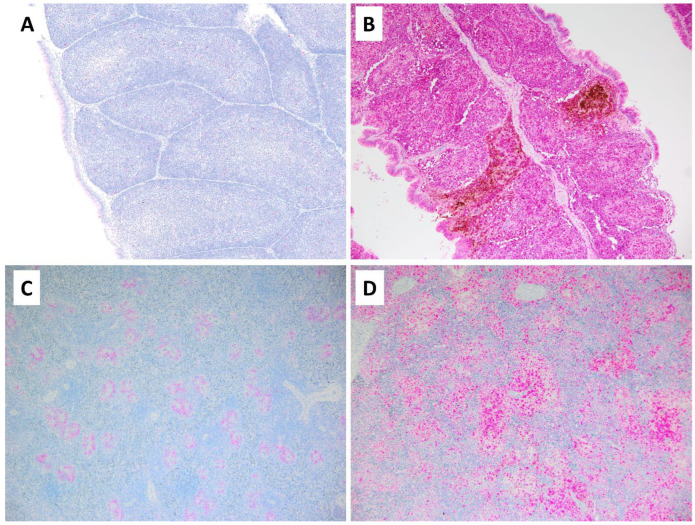
Expression of MLKL. IHC staining of MLKL antigen (red) in uninfected and infected chickens. The bursa of an uninfected control chicken has barely visible red MLKL staining of sparsely dispersed individual lymphocytes, and no indication of viral antigen (**A**). In marked contrast, the bursa of an infected chicken has diffuse MLKL expression in the vast majority of lymphocytes, and staining appears intensely dark red-orange in individual follicles that also are expressing (brown) viral antigen (**B**). The spleen of an uninfected chicken reveals a regular pattern of mild MLKL expression that lies within periarteriolar lymphoid sheaths (**C**), while the spleen of an infected bird shows more extensive expression of MLKL that often bridges between adjacent PALS (**D**).

MLKL protein was detected by IHC in tissues of both uninfected and infected birds; however, the pattern and amount of staining varied between groups (Figure 4). Uninfected birds had mild background levels of MLKL staining of scattered individual lymphocytes in the bursa of Fabricius; in contrast, NDV-infected birds had profoundly intense and diffuse staining for MLKL affecting all bursal follicles. MLKL staining of spleens from uninfected birds revealed a light band of staining restricted to the central portion of periarteriolar lymphoid sheaths (PALSs), while NDV-infected birds had much wider and darker staining with MLKL that often extended and bridged between PALS. The thymuses of uninfected birds had scattered individual lightly MLKL-positive cells, while infected thymuses had large dark patches of MLKL-positive cells. MLKL-positive cells in the kidney were rare in both uninfected and infected birds.

**Figure 5:**
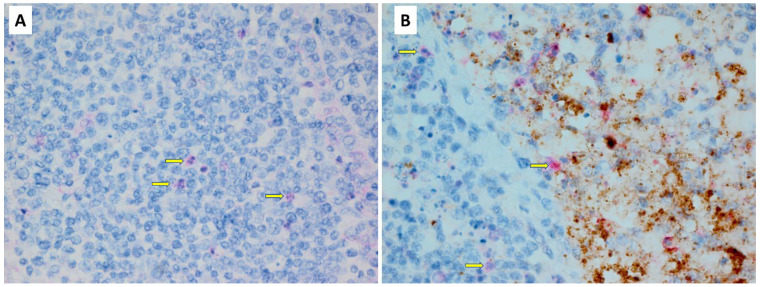
Caspase expression in lymphoid tissues. These high-magnification photomicrographs of the bursae of Fabricius from uninfected (**A**) and NDV-infected (**B**) chickens are largely representative for caspase staining of lymphoid tissues. Minor caspase expression (red stain) is visible in scattered individual cells of both chickens (yellow arrows point to examples). Despite obvious NDV-HN antigen (brown stain) in the infected chicken, caspase expression is not much different from the uninfected chicken.

Only minor differences, if any, for IHC of caspase-3 protein occurred in lymphoid tissues of uninfected and NDV-infected birds, with only modest expression detected in these tissues (Figure 5).

Kidney specimens were often included in paraffin blocks that contained bursae of Fabricius, consequently IHC staining of six kidneys were available for examination as a result of having applied these techniques to bursal tissues. The kidneys of neither uninfected nor infected birds expressed MLKL. The kidneys of uninfected birds showed no expression of caspase-3, but infected birds had nuclear staining of renal tubular epithelial cells, despite the normal histological appearance of the epithelial cells and their nuclei (Supplemental Figure S1). The thymus was only examined in three infected birds, and lymphoid depletion in these was primarily associated with the MLKL marker for necroptosis, but also with a minor increase in caspase-3 expression.

### 3.5. RADD Results

**Figure 6:**
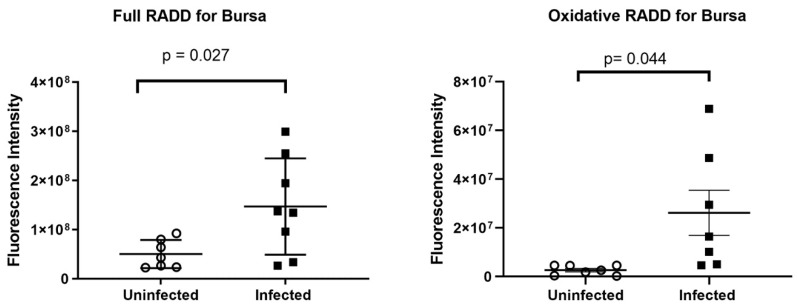
Comparative analysis of the full RADD and Oxidative RADD results in the bursae of Fabricius from uninfected and genotype VII Newcastle disease virus-infected chicken poults. Results are displayed as the mean fluorescence intensity for each tissue. A Student’s *t*-test was used to determine significance as noted.

RADD procedures were sensitive enough to detect normal basal levels of DNA damage in the bursal tissues of the uninfected control chickens. Bursas from uninfected chickens had a mean broad spectrum DNA damage level of 5.0 ± 1.1 × 10^7^ measured by full RADD, with oxidative DNA damage levels at 2.7 ± 0.73 × 10^6^ (*n* = 7) as measured by oxRADD. In comparison, bursal tissue from NDV-infected birds had significant increases in broad spectrum DNA damage levels (1.5 ± 0.35 × 10^8^, *n* = 8, *p* = 0.027) and in oxidative DNA damage (2.6 ± 0.92 × 10^7^, *n* = 7, *p* = 0.044). On average, oxidative lesions accounted for 17% of total DNA damage (Figure 6).

**Figure 7:**
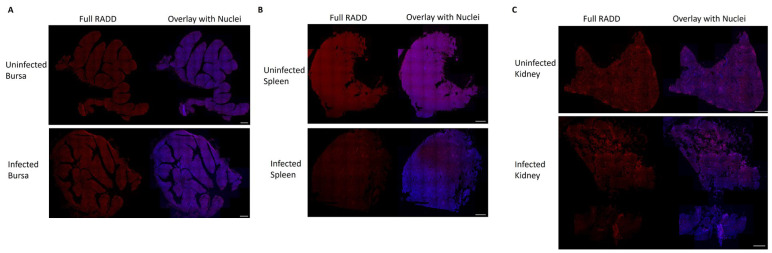
Full RADD assay of DNA damage within tissues of uninfected and infected chicken poults. (**A**) Full RADD signal in the bursa (red) and the overlay of the RADD signal with nuclear staining with Hoechst. (**B**) Full RADD signal in spleen with nuclear overlay. (**C**) Full RADD signal in kidney with nuclear overlay. The scale bar is 500 µm.

As a consequence of performing RADD and oxRADD analyses on the bursa specimens, variable numbers of other tissues (e.g., spleen and kidney) contained in the same paraffin blocks were also subjected to these procedures, allowing us to examine DNA damage in other representative tissues opportunistically but without statistical power. We examined a small cohort of spleens from uninfected control and NDV-infected birds. The full RADD analysis of these spleens showed a higher level of DNA damage within the healthy control birds (1.6 ± 0.19 × 10^8^, *n* = 4) than in the ill NDV-infected birds (6.7 ± 2.2 × 10^7^, *n* = 7) (Figure 7). The oxRADD showed that oxidative lesions only make up a small portion (<2%) of the DNA damage observed in the control birds (2.9 ± 0.090 × 10^6^, *n* = 3), indicating that non-oxidative lesions such as deamination events and crosslinks are more prominent in the control tissues. On average, the infected birds showed seven-fold higher levels of oxidative DNA damage (2.1 ± 0.50 × 10^7^, *n* = 6) in comparison to the uninfected healthy control birds. Oxidative lesions account for approximately 30% of the DNA lesions remaining in the spleens of infected birds (Figure 6).

## 4. Discussion

The viral strains used in this study were even more virulent than anticipated. Chicken poults were inoculated with only 10^2^ EID_50_ of GVII NDV, yet mortality began within 1 day. For humane reasons, all infected birds were euthanized on day 2 after inoculation when the birds were 37 days old, a day earlier than planned. Negative control birds were euthanized as originally scheduled at 38 days of age. Although we acknowledge the 1-day difference in age of the infected and negative control birds, large differences in MLKL and RADD greatly surpass any plausible inherent minor variations that could exist in these parameters between 37- and 38-day-old chickens.

In comparison with the inoculum of virulent NDV-GVII virus used in our experiment (10^2^ EID_50_), Alexander et al. (2006) inoculated poults with 10^6^ EID_50_ NDV-GVI (a 10,000-fold greater comparative dose) and clinical signs did not begin in that study until the second day, with some birds surviving for 4 days [15].

Profound lymphoid depletion was the main lesion induced by infection in our study, and this is in agreement with prior statements about NDV GVII [17]. We sought evidence to distinguish between different types of programmed cell death (apoptosis or necroptosis) and necrosis in various lymphoid organs. Histopathology of the bursa of Fabricius revealed dark condensed pyknotic lymphocyte nuclei, suggestive of programmed cell death. Immunohistology revealed a very high level of expression of MLKL without Caspase-3, which identifies the mechanism of cell death as necroptosis. Histopathological lesions in the spleen of infected birds revealed nuclear changes in lymphocytes ranging between pyknosis and karyorrhexis (nuclear disruption), suggestive of necrosis or of a combination of programmed cell death and necrosis. Spleens also contained fibrinous exudate, indicative of an acute inflammatory process that would be more typical of necrosis than of programmed cell death. Immunohistology of spleens revealed a pattern of increased expression of MLKL in regular narrow bands within PALS, but elsewhere neither MLKL nor caspase-3 had increased expression. These findings in splenic white pulp suggest a larger component of lymphocyte necrosis, admixed with lymphocyte necroptosis in a regional pattern.

From the above observations, we conclude that lymphoid depletion caused by NDV infection results from a combination of necroptosis (a type of non-caspase dependent programmed cell death) and necrosis, with bursal atrophy induced purely by necroptosis. Lymphocyte populations in the spleen are more heterogeneous and include a mixture of mature differentiated cells, in comparison with the bursa and thymus which are expected to contain predominant populations of immature or young B or T cells, respectively. It seems likely that different lymphocyte population subsets are more susceptible to either necroptosis or necrosis, but these responses do not appear to divide simply between B and T cells. Further studies are needed to tease out how the different lymphocyte subpopulations are affected by NDV and why these differences occur.

We sought further information about the depletion of lymphoid organs using repair assisted damage detection (RADD). By analysing a broad spectrum of lesions (full RADD, *p* < 0.03) and the specific levels of oxidative lesions (oxRADD, *p* < 0.05), we observed increased DNA damage within the bursae of Fabricius of infected birds. Viral infection increased the DNA damage load in bursae, and these lesions remained unrepaired, consistent with rapid programmed cell death induced in this tissue, in concordance with the pathological and immunohistochemical findings. The changes observed between the two RADD assays demonstrated that oxidative lesions increased specifically after viral infection. However, the higher full RADD signal shows a significant burden of other DNA lesions, such as deamination events, DNA crosslinks, and strand breaks. NDV infection has previously been shown to upregulate the expression of inducible Nitric Oxide Synthase [18], an example of a pro-oxidant host response that could contribute to oxidative DNA damage.

The full RADD and oxRADD patterns in splenic tissue showed greater complexity, although this tentative observation is based on fewer specimens. It is unclear why non-oxidative damage, such as uracils and crosslinked DNA lesions, were reduced in the spleens of infected birds. This finding may suggest viral upregulation of host DNA damage response pathways in infected birds, which can benefit the replication of RNA viruses [8]. More studies are needed to probe the changes in genomic integrity in the spleen and other tissues. Similar to findings in the bursa of Fabricius, the oxidative repair pathways of infected birds appear to be compromised in the spleen. This pattern of change is consistent with the possibility of necrosis, tending to support the conclusions of histopathology and IHC.

Necroptosis is a well-studied mechanism of cell death in various diseases [19,20,21,22], and is shown in the present study to occur in the bursa of Fabricius, which is primarily composed of immature B lymphocytes. NDV and measles are both paramyxoviruses. Specific studies examining the relationship between measles and necroptosis are not readily available in the literature. Given our findings on necroptosis in the bursa, we suggest that similar studies should be performed for measles, which could potentially uncover new insights into the pathogenesis of measles induced lymphoid depletion.

Mesogenic and velogenic NDV infections cause oxidative stress in the brain, liver [23,24], and bursa of Fabricius [25] of chickens. Our results indicate a significant increase in oxidative stress and broad spectrum DNA damage in bursa of Fabricius induced by NDV infection that is consistent with Kristeen-Teo et al. (2017) [25].

The renal tubular epithelium of NDV-infected poults were the only cells that showed a substantial increase in expression of caspase-3, nevertheless the histological appearance of the kidney was usually normal. These findings may be evidence of circulatory shock leading to early renal hypoxia and the onset of caspase expression. There was concurrent uneven distribution of viral antigen within the renal cortex as detected by IHC, so more direct viral mechanisms of apoptosis induction could also be considered.

## 5. Conclusions

This study of Newcastle disease virus infection in chickens convincingly demonstrates that the mechanism responsible for rapid and profound bursal atrophy of B lymphocytes involves necroptosis rather than apoptosis, at least for genotype VII strains, which is a novel observation. Increased necroptosis also appears to occur in the thymus, but depletion of splenic white pulp appears to result primarily from necrosis with a regularly distributed lesser component of necroptosis. We also report the first use of repair assisted DNA damage (RADD) and oxidative RADD procedures for the study of ND pathogenesis, which reveals increases in oxidative DNA damage in both the bursa and spleen, but possibly with a curious decrease in non-oxidative DNA damage in spleens of infected birds. Because NDV and lymphoid depletion in Newcastle disease is similar to lymphoid depletion in other paramyxoviral diseases, we suggest that necroptosis and DNA damage repair patterns should also be examined for measles.

## Figures and Tables

**Table 1:** Antibodies used for immunohistochemistry.

Antibody	Clone	pH	Dilution
Anti-Newcastle disease virus antibody	8H2, mouse, monoclonal	9.0	1:500
Anti-caspase-3 antibody ab4051	Rabbit polyclonal	6.0 and 9.0	1:250
Anti-MLKL antibodyMABC604	3H1 mouse monoclonal	7.4	1:500

**Table 2:** RADD sequential reaction conditions. RADD is performed in two sequential reactions without aspirating reagents between reactions. The lesion processing mix (left) is placed on prepared tissues and placed in a humidified incubator. The gap filling mix (right) is added directly to the lesion processing mix and incubated for an additional hour. The reagents are then aspirated, and the cells are washed and incubated with anti-digoxigenin antibody.

Lesion Removal Mix	100 µL Total Reaction Volume	Gap Filling Mix	100 µL Total Reaction Volume
UDG (NEB M0280)	2.5 U	Klenow exo^−^ (Thermo Fisher EP0422)	1.0
FPG (NEB M0240)	4 U	Digoxigenin dUTP (Sigma Aldrich 11093088910)	0.1
T4 PDG (NEB M0308)	5 U	Thermo Pol Buffer (NEB B9004)	10 µL
EndoIV (NEB M0304)	5 U		
EndoVIII (NEB M0299)	5 U		
NAD^+^ (100×, NEB B9007)	500 µM		
BSA (Sigma Aldrich)	200 µg/mL		
Thermo Pol Buffer (NEB B9004)	10 µL		

## Data Availability

The original contributions presented in the study are included in the article/Supplementary Materials, further inquiries can be directed to the corresponding authors.

## Supplementary Materials

The following supporting information can be downloaded at: https://www.mdpi.com/article/10.3390/pathogens13080619/s1, Figure S1: Immunohistochemistry for caspase-3 antigen (red stain) in kidneys. (A) Kidney from an uninfected bird. There is no evidence of caspase-3 expression. (B) Kidney from an infected bird. Profiles of some renal tubules have red staining of epithelial nuclei (yellow arrow points to an example), indicative of caspase-3 expression.
