# Supplementary figures and images for: Newcastle Disease Virus Induces Profound Lymphoid Depletion with Different Patterns of Necroptosis, Necrosis, and Oxidative DNA Damage in Bursa, Spleen, and Other Lymphoid Tissues
Source: Pathogens. 2024 Jul 26;13(8):619. doi: 10.3390/pathogens13080619 (PMC11357213; doi:10.3390/pathogens13080619)

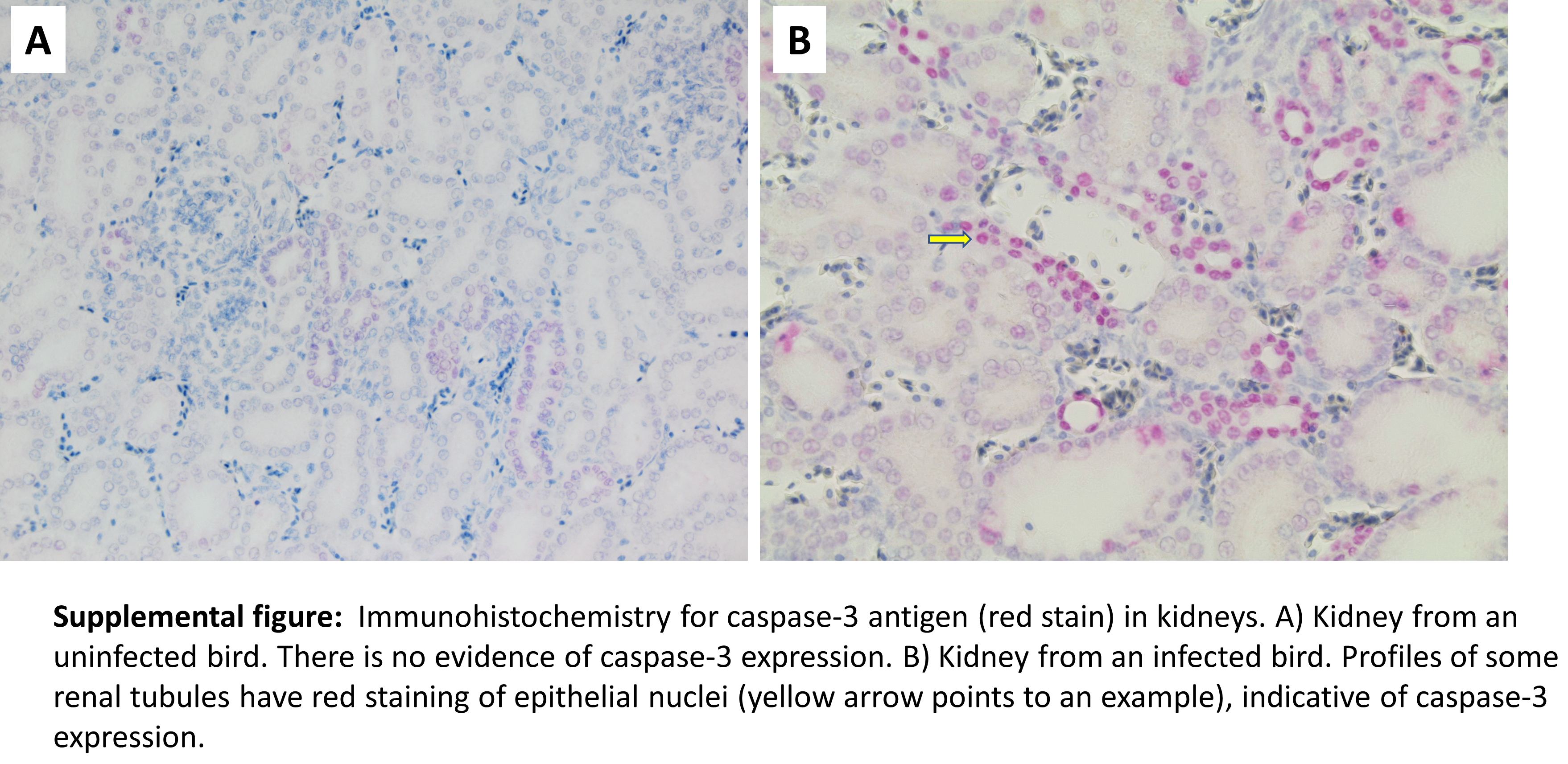

Supplement: Supplementary file 1 [file pathogens-13-00619-s001.zip › pathogens-3038835-supplementary.tif]
